# Transient inflammation-induced ongoing pain is driven by TRPV1 sensitive afferents

**DOI:** 10.1186/1744-8069-7-4

**Published:** 2011-01-10

**Authors:** Alec Okun, Milena DeFelice, Nathan Eyde, Jiyang Ren, Ramon Mercado, Tamara King, Frank Porreca

**Affiliations:** 1 Department of Pharmacology, College of Medicine, University of Arizona, Tucson, AZ 85724, USA

## Abstract

**Background:**

Tissue injury elicits both hypersensitivity to evoked stimuli and ongoing, stimulus-independent pain. We previously demonstrated that pain relief elicits reward in nerve-injured rats. This approach was used to evaluate the temporal and mechanistic features of inflammation-induced ongoing pain.

**Results:**

Intraplantar Complete Freund's Adjuvant (CFA) produced thermal hyperalgesia and guarding behavior that was reliably observed within 24 hrs and maintained, albeit diminished, 4 days post-administration. Spinal clonidine produced robust conditioned place preference (CPP) in CFA treated rats 1 day, but not 4 days following CFA administration. However, spinal clonidine blocked CFA-induced thermal hyperalgesia at both post-CFA days 1 and 4, indicating different time-courses of ongoing and evoked pain. Peripheral nerve block by lidocaine administration into the popliteal fossa 1 day following intraplantar CFA produced a robust preference for the lidocaine paired chamber, indicating that injury-induced ongoing pain is driven by afferent fibers innervating the site of injury. Pretreatment with resiniferatoxin (RTX), an ultrapotent capsaicin analogue known to produce long-lasting desensitization of TRPV1 positive afferents, fully blocked CFA-induced thermal hypersensitivity and abolished the CPP elicited by administration of popliteal fossa lidocaine 24 hrs post-CFA. In addition, RTX pretreatment blocked guarding behavior observed 1 day following intraplantar CFA. In contrast, administration of the selective TRPV1 receptor antagonist, AMG9810, at a dose that reversed CFA-induced thermal hyperalgesia failed to reduce CFA-induced ongoing pain or guarding behavior.

**Conclusions:**

These data demonstrate that inflammation induces both ongoing pain and evoked hypersensitivity that can be differentiated on the basis of time course. Ongoing pain (a) is transient, (b) driven by peripheral input resulting from the injury, (c) dependent on TRPV1 positive fibers and (d) not blocked by TRPV1 receptor antagonism. Mechanisms underlying excitation of these afferent fibers in the early post-injury period will offer insights for development of novel pain relieving strategies in the early post-traumatic period.

## Background

Tissue injury elicited by trauma or disease produces pain that is often described as dull, aching, throbbing and ongoing. Additionally, injury produces long-lasting tenderness at and surrounding the injury site that is reflected by pain resulting from hypersensitivity to external stimuli such as touch or movement. While mechanisms underlying hypersensitivity to evoked stimuli have been extensively studied preclinically, understanding ongoing (i.e., non-evoked) pain in animal models has been more difficult.

Ongoing pain, i.e., pain that is not "evoked" is an important part the human pain experience, particularly in the early periods following tissue damage such as might occur in the post-operative state. Clinical and preclinical reports indicate that such pain could be mechanistically distinct from processes mediating evoked hyperalgesia and/or allodynia [1-3]. Until recently, limitations in approaches for measurement of spontaneous or ongoing pain in animal models have prevented detailed mechanistic explorations of such pain [1,4,5]. We have recently shown that relief of experimental neuropathic pain produces negative reinforcement, demonstrated by place preference for a chamber paired with pain relief, unmasking spontaneous pain [1]. Here, we determined whether this measure could be applied to other pain conditions such as inflammatory pain and to determine possible mechanisms underlying acute injury-induced pain.

Several models for injury-induced pain have been developed including hindpaw injection of complete Freund's adjuvant (CFA), a widely used model of persistent inflammatory pain [6-9]. CFA produces thermal and mechanical hypersensitivity lasting for several weeks following administration into the hindpaw [7-9]. Additionally, CFA elicits time-dependent spontaneous activity of primary afferent fibers [6,10] that may underlie injury-induced pain [6,10-13]. The mechanisms underlying ongoing, unprovoked pain, as might correspond clinically to pain at rest, are not well understood [5,13]. Here, we characterize the time-dependent expression of injury-induced pain following hindpaw CFA-induced inflammation, comparing measures of evoked pain, guarding behavior, and conditioned place preference to pain relief. Further, we begin to delineate specific afferent subtypes that may drive CFA-induced pain.

## Results

### Injury-induced evoked and ongoing pain

**Figure 1:**
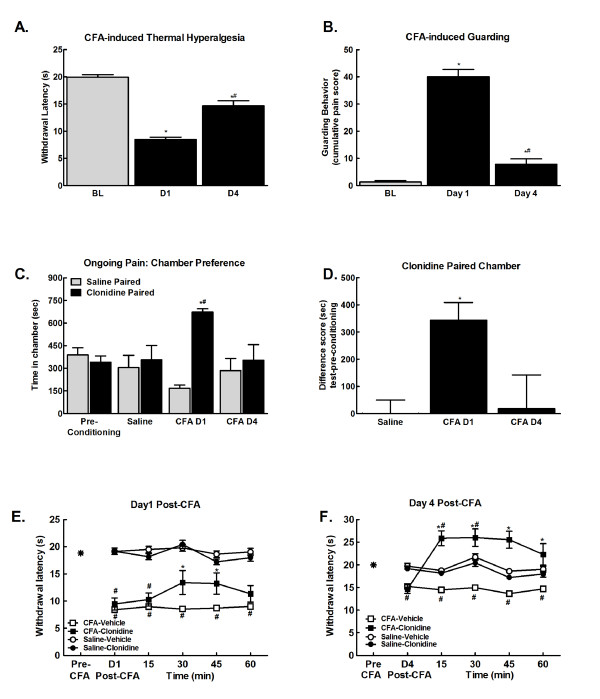
**A. Hindpaw CFA produced lower paw withdrawal latencies within 24 hours (D1)**. Paw withdrawal latencies remained lower than pre-injury baseline (BL) 4 days following injury, but were higher than those observed 1 day following injury. *p < 0.05 vs. pre-injury thresholds (BL); ^#^p < 0.05 vs. D1 thresholds, n = 7-8. B. Hindpaw CFA produced guarding behavior within 24 hours (D1). Guarding behavior remained elevated compared to pre-injury baselines (BL) 4 days following injury, but was significantly reduced compared to the D1 time-point. *p < 0.05 vs. pre-injury thresholds (BL); ^#^p < 0.05 vs. D1 thresholds, n = 7-8. C. Spinal clonidine (10 μg) induced CPP selectively in CFA treated rats at day 1, but not day 4 post CFA, injection. No chamber preference is observed in saline treated rats indicating that this dose of spinal clonidine is not rewarding in the absence of injury. *p < 0.05 vs. saline paired chamber; ^#^p < 0.05 vs. pre-conditioning, n = 7. D. Difference scores calculated as test time - preconditioning time spent in clonidine chamber confirm that CFA treated rats showed CPP 24 hrs, but not 4 days post CFA injection. *p < 0.05 vs. saline treated group. E. CFA decreased paw withdrawal latencies to radiant heat within 24 hours of injection (Post-CFA). Spinal clonidine (10 μg) attenuated, but did not fully reverse CFA-induced thermal hyperalgesia. This dose of spinal clonidine failed to induce antinociception in rats that received intraplantar saline, *p < 0.05 vs. post-CFA, ^#^p < 0.05 vs. pre-CFA, n = 5-8. F. CFA-induced thermal hyperalgesia persisted through day 4 post-CFA. Spinal clonidine (10 μg) induced antihyperalgesia and antinociception, elevating paw withdrawal latencies above baseline values. *p < 0.05 vs. post-CFA, ^#^p < 0.05 vs. pre-CFA, n = 5-8.

Consistent with previous studies, CFA produced thermal hypersensitivity within 24 hrs that lasted through 4 days post injection, with an apparent time-dependent decrease in injury-induced thermal hypersensitivity (Figure 1a, *p < 0.05 vs. BL, ^#^p < 0.05 vs. D1). Comparison of difference scores confirms that there is a significant decrease in the CFA-induced thermal hypersensitivity across days. CFA induced a 10.6 ± 0.7 s change from BL at D1 that decreased to a 5.2 ± 0.96 s change from BL at D4 (p < 0.01, paired t-test).

CFA induced guarding behavior within 24 hours that is diminished by 4 days post-injury (Figure 1b, ***p < 0.01 vs. BL; *p < 0.05 vs. BL). These findings are consistent with the time-course of guarding behavior reported with CFA [6].

In initial experiments, CFA-treated rats received spinal saline both in the morning and afternoon sessions 1 day following CFA injection as a control for possible CFA-induced aversion to the morning chamber (data not shown). No chamber preferences were observed in these animals on test day, with times of 411.2 ± 99.7 s in the morning saline paired chamber and 406.1 ± 100.9 s spent in the afternoon saline paired chamber. These data indicate that introducing the CFA-induced pain 20 hrs prior to the start of chamber pairing (conditioning day) did not produce an aversion to the morning paired chamber.

The possible presence of CFA-induced ongoing pain and its time course was then determined using conditioned place preference (CPP) to spinal clonidine. No pre-conditioning chamber differences were observed any of the treatment groups, therefore all pre-conditioning data were pooled for graphical representation. (Figure 1c,). Following the habituation period on the third day, one group of rats received i.paw CFA or saline (CFA D1). Administration of spinal clonidine 1 day following CFA injection produced robust chamber preference (Figure 1c; *p < 0.05 vs. pre-conditioning). To determine if ongoing pain persisted across 4 days, a separate group of rats received i.paw CFA or saline 1 day prior to the 3 day habituation period (CFA D4). Administration of spinal clonidine 4 days following CFA injection failed to induce chamber preference (Figure 1c; *p < 0.05 vs. pre-conditioning). Rats that received intraplantar saline failed to show preference for the clonidine paired chamber irrespective of whether saline was administered 24 hrs or 4 days prior to conditioning day (p > 0.05). Therefore, all saline data were pooled for graphical representation. Difference scores confirmed that CFA treated rats demonstrated conditioned place preference to the clonidine paired chamber 1 day, but not 4 days following CFA injection (Figure 1d; *p < 0.05 vs. saline).

Spinal administration of clonidine attenuated CFA-induced thermal hyperalgesia 1 day following CFA injection (Figure 1e, ^#^p < 0.05 vs. pre-CFA; *p < 0.05 vs. post-CFA). Clonidine fully reversed CFA-induced thermal hyperalgesia at the D4 time-point, and produced antinociception, with paw-flick latencies significantly higher than pre-CFA baseline values (Figure 1f, ^#^p < 0.05 vs. pre-CFA; *p < 0.05 vs. post-CFA) This dose of spinal clonidine failed to alter paw-flick latencies of control rats 1 or 4 days following intraplantar saline (Figure 1e,f).

### Ongoing pain is driven by peripheral input

**Figure 2:**
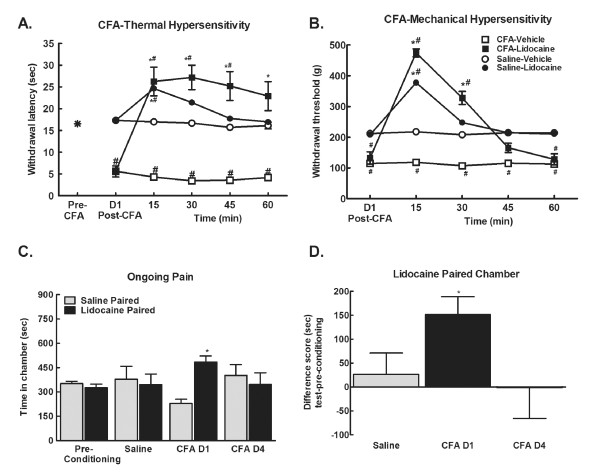
**A. CFA reduced withdrawal latency within 24 hrs post-CFA**. Popliteal fossa lidocaine (4% w/v, 200 μl) reversed CFA-induced hypersensitivity, elevating paw withdrawal latencies to noxious thermal stimulation to almost cut-off in both CFA and control (saline) treated rats at the 15 min time-point. Elevated paw-flick latencies were observed throughout the 60 min testing period in the CFA treated rats, and returned to pre-lidocaine latencies within 45 min in the control rats, *p < 0.05 vs. post-CFA, ^#^p < 0.05 vs. pre-CFA, n = 5-6. B. CFA decreased withdrawal thresholds to noxious mechanical stimulation within 24 hrs. Popliteal fossa lidocaine elevated paw-withdrawal thresholds to almost cut-off in both the CFA and saline (control) groups at the 15 min time-point. Paw-withdrawal thresholds returned to pre-lidocaine values within 30 min for control rats, and within 45 min in the CFA treated rats, *p < 0.05 vs. post-CFA, ^#^p < 0.05 vs. pre-CFA, n = 5-6. C. Popliteal fossa lidocaine induced CPP 24 hrs, but not 4-days post-CFA injection. *p < 0.05 vs. pre-conditioning, p < 0.05, n = 5-6. D. Difference scores calculated as test time - preconditioning time spent in lidocaine chamber confirm that only rats treated with CFA 24 hours prior to conditioning showed CPP in response to peripheral nerve block by lidocaine. *p < 0.05 vs. saline treated group.

Administration of lidocaine into the popliteal fossa 24 hrs following intraplantar CFA or saline produced a nerve block as indicated by an essentially complete blockade of responses to noxious thermal or mechanical stimuli in saline and CFA-treated rats. Paw withdrawal latencies to the noxious thermal stimulus were extended almost to cut-off following lidocaine injection (Figure 2a, ^#^p < 0.05 vs. pre-CFA; *p < 0.05 vs. post-CFA values). Similarly, administration of lidocaine into the popliteal fossa raised paw withdrawal thresholds evaluated with the Randall-Sellito test to near cut-off levels in both CFA- and saline-pretreated rats (Figure 2b, ^#^p < 0.05 vs. saline; *p < 0.05 vs. post-CFA values). This dose of lidocaine clearly impaired motor function in both CFA and saline (control) treated rats determined by visual inspection of locomotion. Motor function was fully recovered in these rats within 2 hrs of lidocaine injection.

To determine the role of injury-induced peripheral drive ongoing pain, animals were treated with lidocaine or saline (control) into the popliteal fossa on conditioning day, 24 hrs following intraplantar administration of CFA or saline. No pre-conditioning chamber differences were observed across any condition, therefore data were pooled for graphical representation (Figure 2c). On test day, 20-24 hrs following conditioning, animals were tested for chamber preference in the absence of popliteal fossa lidocaine, eliminating potential effects of motor impairment on chamber exploration. Rats treated with CFA 24 hours prior to conditioning demonstrated preference for the chambers paired with popliteal fossa lidocaine. Rats that received CFA 4 days prior to conditioning and the saline treated rats did not show preference (Figure 2c, *p < 0.05 vs. pre-conditioning). Comparison of difference from baseline scores confirm that rats treated with CFA 24 hrs prior to conditioning increased time spent in the lidocaine paired chambers following conditioning whereas rats treated with CFA 4 days prior to conditioning did not (Figure 2d, *p < 0.05 vs. saline).

### CFA-induced evoked and ongoing pain is dependent on TRPV1 positive fibers

**Figure 3:**
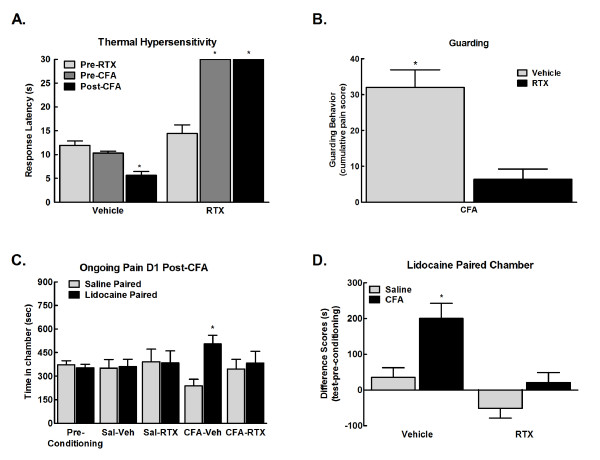
**A. RTX (0.1 mg/kg, i.p.) treatment 4 days prior to testing blocked thermal nociception with all responses on the hot plate (52°C) reaching the cut-off time (32 s)**. CFA reduced the thermal response latency within 24 hr in animals that received vehicle for RTX. Thermal hypersensitivity was completely blocked in the RTX treated group. *p < 0.05 vs. pre-RTX; ^#^p < 0.05 vs. vehicle, n = 7. B. RTX treatment 4 days prior to testing blocked CFA induced guarding of the hindpaw observed 24 hours following injury. *p < 0.05 vs. vehicle treated, n = 8-10. C. RTX treatment 1 day prior to testing prior to habituation blocked CPP to chambers paired with lidocaine administration into the popliteal fossa in CFA treated rats 24 hr post-CFA. Rats that received saline into the hindpaw failed to show preference for the lidocaine paired chamber irrespective of RTX treatment. *p < 0.05 vs. pre-conditioning, n = 6-8. D. Difference scores calculated as test time - preconditioning time spent in lidocaine chamber confirm that only CFA treated rats that did not receive RTX (Vehicle) showed preference for the lidocaine paired chamber. *p < 0.05 vs. saline treated group.

Systemic administration of RTX has been demonstrated to produce long-lasting desensitization of TRPV1-expressing nociceptors [14] and to essentially eliminate sensitivity to noxious thermal stimuli in animals [15]. Rats treated with RTX failed to respond to a noxious thermal stimulation (Figure 3a, *p < 0.05 vs. pre-RTX). Separate groups of rats were observed for guarding behavior 24 hours following CFA. Systemic administration of RTX 3 days prior to injury blocked the injury-induced guarding behavior (Figure 3b, *p < 0.05 vs. vehicle). RTX administration prior to habituation blocked CPP resulting from administration of lidocaine into the popliteal fossa in CFA treated rats (Figure 3c, *p < 0.05 vs. pre-conditioning). Rats treated with i.paw saline (controls) did not show preference for the lidocaine paired chamber irrespective of RTX treatment. Difference from baseline values confirm that CFA treated rats that did not receive RTX (vehicle) showed increased time in the lidocaine paired chambers whereas CFA treated rats that received RTX treatment did not show increased time spent in the lidocaine paired chamber (Figure 3d, *p < 0.05 vs. vehicle).

### CFA-induced evoked, but not ongoing pain is dependent on TRPV1 receptors

**Figure 4:**
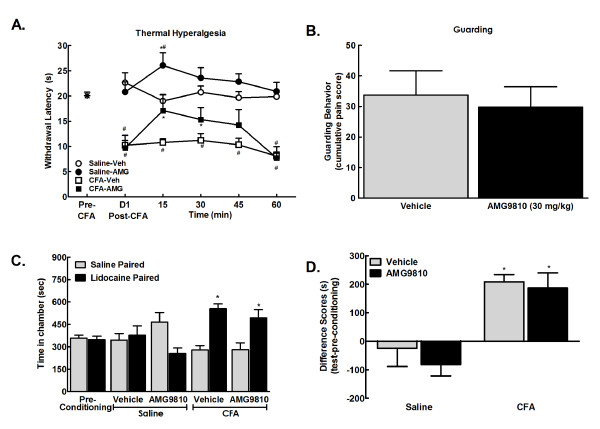
**A. CFA reduced withdrawal latency within 24 hrs post-CFA**. AMG9810 (30 mg/kg, i.p.) reversed CFA-induced thermal hyperalgesia within 15 min, with paw-flick latencies returning to pre-drug levels 60 min post administration. AMG9810 also elevated paw-flick latencies of control (saline treated) rats with 15 min of administration. *p < 0.05 vs. post-CFA, ^#^p < 0.05 vs. pre-CFA, n = 5-6. B. Systemic administration of the selective TRPV1 receptor antagonist, AMG9810 (30 mg/kg i.p.) failed to alter CFA-induced guarding behavior 24 hours following injury, p > 0.05 vs. vehicle. C. Pre-conditioning values did not differ between treatment groups, therefore values were pooled for graphical representation. Lidocaine increased time spent in the lidocaine paired chambers in both the AMG9810 and vehicle treated rats, *p < 0.05 vs. pre-conditioning, n = 5. D. Difference scores calculated as test time - preconditioning time spent in the lidocaine chamber confirm that CFA treated rats increased time spent in the lidocaine paired chambers in both the vehicle and AMG9810 treatment groups, *p < 0.05 vs. saline/vehicle.

In agreement with previous reports [16], systemic administration of the TRPV1 receptor AMG9810 (30 mg/kg, i.p.) blocked CFA-induced thermal hypersensitivity 1 day post CFA injection (Figure 4a, *p < 0.05 vs. post-CFA, ^#^p < 0.05 vs. pre-CFA).

To determine whether selective blockade of the TRPV1 receptor blocks CFA-induced pain, rats received systemic administration AMG9810 (30 mg/kg, i.p.) and were observed for guarding behavior 24 hours following injury. Administration of the TRPV1 antagonist failed to block guarding behavior in CFA treated rats (Figure 4b).

To determine whether selective blockade of the TRPV1 receptor blocks injury induced ongoing pain, rats underwent single trial CPP to popliteal fossa lidocaine in the presence of AMG9810 (30 mg/kg, i.p.) or vehicle 24 hours following CFA administration. No pre-conditioning chamber differences were observed across any condition, therefore data were pooled for graphical representation (Figure 4c). Rats received hindpaw injection of saline or CFA immediately following baseline assessment. On conditioning day, rats received systemic administration of PEG400 (vehicle for AMG9810) 30 min before popliteal fossa administration of saline and placed into a pre-determined pairing chamber. Four hours later, rats received AMG9810 (30 mg/kg, i.p.) or PEG400 30 min before popliteal fossa administration of lidocaine and placed into the opposite pairing chamber. Following conditioning, CFA treated rats showed CPP to the lidocaine paired chamber irrespective of whether they received AMG9810 (Figure 4c, *p < 0.05 vs. pre-conditioning values). Difference scores confirmed that AMG9810 failed to block popliteal fossa lidocaine induced CPP (Figure 4d, *p < 0.05 vs. saline-vehicle).

## Discussion

The present study provides a direct demonstration that hindpaw injury elicits transient injury-driven ongoing pain that is temporally distinct from long-lasting evoked hypersensitivity. The temporal dissociation between injury-induced ongoing pain and evoked hypersensitivity supports previous suggestions of mechanistic differences in these components of inflammatory pain [12,13,17,18]. Additionally, the data indicate that ongoing pain induced by inflammation injury (CFA) is dependent upon input from TRPV1 positive afferent fibers, likely driven from the injury. Our data indicate that CFA-induced injury provides a tonic aversive stimulus that persists for at least one day post-injury and that relief from this aversive state is sufficient to induce reward, consistent with negative reinforcement. Pain relief was induced by spinal clonidine or peripheral nerve block, manipulations at sites that do not directly activate the reward pathway. These data are the first to demonstrate reward in animals by peripheral nerve block following injury.

Consistent with many previous reports, this study confirmed that CFA induced long-lasting hypersensitivity to acute application of both noxious and non-noxious (tactile) stimulation [7-9,19,20]. Whether, and when, such injuries might elicit ongoing pain, however, was not definitively known. Lawson and colleagues detected time-dependent foot-lifting behaviors at day 1 following CFA but this behavior was virtually absent by post-CFA day 4 [6]. However, whether such behaviors reflect ongoing pain has been questioned [12]. Treatment with CFA produce clear evoked hypersensitivity at day 1, which diminishes within 4 days, suggestive of more intense "pain" at day 1 than at later time points. A high degree of evoked hypersensitivity might contribute to avoidance of contact with surfaces, resulting in foot-lifting behaviors [12]. Previous studies have demonstrated that CFA induces mechanical sensitization in both A- and C-fibers as well as other indicators of neuronal sensitization including spontaneous activity and expanded receptive fields within 24 hrs of CFA injection [20]. Moreover, the study of Lawson and colleagues revealed that CFA induced a significant (>25%) increase in percentages of C- and Aδ fibers that showed spontaneous discharge both at day 1 and day 4 post-CFA, in spite of differences in foot-lifting behaviors at these points [6]. Work by Xiao and Bennett has demonstrated that CFA treatment induces spontaneous discharge of C-fibers that are similar across days 2-7 post-treatment and that additionally, both the frequency and prevalence of such spontaneous afferent activity is very low making it uncertain whether this level of afferent drive might elicit ongoing pain [10].

In the present studies, we demonstrated CPP following spinal clonidine or lidocaine injection into the popliteal fossa (local nerve block) at 24 hrs, but not 4 days following injection, suggesting the transient presence of ongoing pain in this model. Spinal clonidine did not induce CPP in non-CFA treated rats indicating that this dose of spinal clonidine did not elicit reward in the absence of pain. The lack of clonidine- or peripheral lidocaine- induced CPP at post-CFA day 4 suggests that injury induced ongoing pain is either absent, or greatly diminished, so that any possible pain relief elicits insufficient negative reinforcement that is detectable in the CPP paradigm. A previous study has explored whether CFA-induced inflammation may represent an aversive stimulus that might be detectable with CPP following administration of drugs for pain relief [18]. In that study, however, a clear demonstration of negative reinforcement was not achieved, likely due to differences in route and timing of administration of pain relieving drugs and other differences in experimental conditions.

The conclusion of transient ongoing pain with CFA-induced inflammation is consistent with clinical knowledge indicating that pain is maximal immediately following injury and diminishes with time. Injuries associated with peripheral inflammation (e.g., surgery) elicit initial transient "spontaneous" pain (driven by the injury) followed by much longer lasting evoked hypersensitivity (i.e., tenderness) of the injured area [21-25]. The characteristics of CFA-induced pain have been documented in a human report [26]. In this report, the consequences of accidental CFA injection into the third digit of the left hand elicited spontaneous "throbbing" pain across the first 24-30 hrs that had diminished within 48 hrs and that was extinct by 7 days [26]. In spite of the dissipation of throbbing pain, evoked hypersensitivity persisted for many weeks [26].

Studies by Brennan and colleagues have used a plantar incision model to demonstrate the presence of time-dependent guarding behaviors following plantar incision that involves skin, fascia and underlying muscle [13]. Guarding behaviors were more modest in the absence of incision of muscle tissue suggesting a strong contribution of nociceptive afferents from this tissue in driving ongoing pain. Increased spontaneous activity of muscle nociceptors was observed one day after skin and deep tissue injury that returned to control levels by 7 days post-incision, corresponding to measures of guarding behaviors [13]. These studies suggested a requirement for afferent drive from deep tissue in the generation of ongoing pain following incision injury. This conclusion appears consistent with the current behavioral observation of transient ongoing pain following CFA. Subcutaneous CFA produces an immune response in the connective tissue sheath that covers the muscle as well as within the muscle itself and this may therefore elicit spontaneous discharge and sensitization of muscle afferents [10]. In our studies, peripheral nerve block with infiltration of lidocaine within the popliteal fossa was demonstrated to produce CPP only in rats with hindpaw inflammation on post-injury day 1. The demonstration of reward following pain relief by peripheral nerve block indicates that afferent drive, likely from deep muscle nociceptors, provides a significant and ongoing aversive stimulus. This aversive stimulus likely mimics the transient features of the inflammatory state associated with trauma, e.g., such as post-operative pain.

Nerve block by local anesthetic injection upstream of tissue injury replicates routine clinical practice to produce relief of pain [27]. It is important to note that CPP was observed following popliteal fossa lidocaine only in injured rats, supporting the conclusion that CFA pain is aversive, driven by primary afferent fibers, and that relief of such pain by nerve block is sufficient to elicit reward. Administration of lidocaine into the popliteal fossa was confirmed to produce an effective nerve block as demonstrated by thermal and mechanical response thresholds that were raised to near cut-off levels in both CFA and saline-treated rats. Demonstration of CPP by a peripheral manipulation such as nerve block has not previously been reported but this result would be predicted if there was negative reinforcement arising from relief of an ongoing aversive stimulus, in this case resulting from injury-induced increased in input from nociceptive afferent fibers.

The TRPV1 receptor is a signal transduction channel primarily located on small to medium diameter primary afferent fibers that responds to noxious thermal stimulation and to acidic environments [28]. This receptor has been demonstrated to play an important role in inflammation injury induced evoked pain [16,29]. Here, we produced prolonged desensitization of TRPV1 positive fibers using resiniferatoxin (RTX) [30]. Systemic administration of RTX has been demonstrated to induce long-lasting insensitivity to thermal stimulation while leaving nociceptive mechanical thresholds unaltered [15,30]. Pretreatment with RTX eliminated responses to noxious thermal stimuli in CFA-treated rats consistent with previous observations in other injury models [19].

In the present studies, CFA-induced ongoing pain was abolished by desensitization of TRPV1 positive afferent fibers following pretreatment with RTX. This result suggests that this subpopulation of nociceptive afferents is responsible for the aversive nature of ongoing pain following hindpaw inflammation. This conclusion is consistent with data from studies of incisional pain. Brennan and colleagues have shown that local infiltration, or perineural administration of capsaicin decreased guarding behaviors as well as afferent spontaneous activity supporting a requirement for TRPV1 positive fibers in ongoing pain resulting from plantar incision [31,32]. Consistent with these findings, RTX treatment blocked CFA-induced guarding behavior in rats. In contrast, selective blockade of the TRPV1 receptor with an antagonist failed to block guarding behavior induced by CFA. This is consistent with other reports demonstrating that selective blockade of the TRPV1 receptor at a dose sufficient to block thermal hypersensitivity failed to block incision-induced guarding behavior [33]. Our studies are consistent with these conclusions in that blockade of the TRPV1 channel failed to block ongoing pain. Here, CPP was elicited by peripheral nerve block regardless of blockade of the TRPV1 receptor. Thus, manipulations that selectively block function of TRPV1 positive fibers are sufficient to block ongoing pain, guarding behavior, and thermal hypersensitivity whereas selective blockade of the TRPV1 channel alone is insufficient to block injury-induced ongoing pain. These data suggest that other, non-TRPV1 targets on TRPV1 positive fibers are mediating injury-induced ongoing pain.

## Conclusions

The current study demonstrates that injury induces a transient state of ongoing pain that is temporally and mechanistically distinct from evoked hypersensitivity. Detailed investigation of mechanisms driving ongoing versus evoked pain may prove essential in development of effective drugs targeting different aspects of clinically relevant pain. CFA induced ongoing pain is driven by afferent input, specifically TRPV1 positive nociceptive fibers. Thus, targeted disruption of injury-induced mediators driving this class of afferent fibers may prove important in providing effective pain relief for patients with ongoing acute pain associated with injury accompanied by inflammation as epitomized by pain at rest in early post-surgical periods. Such therapies would lack central effects associated with opioids, currently the most commonly used post-operative therapy.

## Materials and methods

### Animals

Male Sprague-Dawley rats (Harlan, Indianapolis, IN) weighing 250-275 g were used in all studies. All procedures involving animals were reviewed and approved by the Institutional Animal Care and Use Committee of the University of Arizona, and were in accord with the guidelines established by the National Institutes of Health.

### Surgical Procedures

Rats were anesthetized with isofluorane and intrathecal catheters were implanted as previously detailed [34]. The atlanto-occipital membrane was exposed, an incision was made in the dura mater, and PE-10 tubing was advanced 8 cm caudally to the lumbar spinal cord. The tubing was exteriorized, filled with saline and plugged with wire. The wound was closed, and animals allowed to recover for 7 days. Notably, baseline testing prior to the 7 day recovery period results in limited chamber crossings and resultant chamber bias in the place preference apparatus, likely due to the invasiveness of the surgery.

### Induction of injury

Rats received intraplantar injection of complete Freund's adjuvant (CFA) (100 μl, s.c.; Calbiochem) into the left hindpaw. Control rats received an equivolume saline injection.

### Drug administration

#### Spinal administration

Clonidine (10 μg; Tocris Bioscience) or saline (vehicle control) was delivered through an intrathecal catheter in a 5 μl volume followed by a 9 μl saline flush. Progress was monitored with an air bubble.

#### Popliteal fossa administration

Lidocaine (4% w/v; Roxane Laboratories) or saline (vehicle control) was delivered in a 200 μl volume. Pilot studies with dye injections confirmed that this volume successfully covered sciatic nerve and included the branches of the sciatic nerve at the bifurcation including the common peroneal, sural, and tibial nerves located within the popliteal fossa [35].

#### Systemic drug administration

The ultrapotent TRPV1 receptor agonist, resiniferatoxin (RTX, Tocris Bioscience), was dissolved in 99.1% saline, 0.3% Tween 80, and 0.6% Ethanol (used as a vehicle control). RTX was administered systemically (0.1 mg/kg, i.p.) in a dose previously demonstrated to eliminate thermal responses across a period of 40 days, the longest time-point tested [15]. RTX was delivered 4 days prior to testing of evoked pain or 1 day prior to habituation for the CPP procedure, a time corresponding to 4 days prior to conditioning day. The TRPV1 receptor antagonist, AMG9810, was dissolved in PEG400 (used as the vehicle control). AMG9810 was administered systemically at a dose (30 mg/kg, i.p.) previously demonstrated to effectively block CFA-induced thermal and tactile hypersensitivity [16].

### Behavioral observations

#### Thermal antinociception and hypersensitivity

Nociceptive withdrawal thresholds to noxious radiant heat were determined using the plantar test apparatus (Ugo Basile, Comerio, Italy) as previously described [15]. A maximal cut-off time of 32 s was used to prevent tissue damage. Elimination of thermal responsiveness in rats treated with RTX was determined with a hotplate (Columbus Instruments) set at 52°C. Latency to the first escape response (jumping, licking, or climbing) was recorded.

#### Mechanical hypersensitivity

Withdrawal thresholds to noxious mechanical stimulation were determined using a Randall-Selitto apparatus (Ugo Basile, Comerio, Italy). A positive response was indicated by withdrawal of the paw. A cut-off of 400 g was used to avoid tissue injury.

#### Tactile hypersensitivity

Paw withdrawal thresholds were determined in response to probing with calibrated von Frey filaments (Stoelting, Wood Dale, IL) using the "up and down" method and analyzed using a Dixon nonparametric test [36,37].

#### Guarding behavior

Assessment of guarding behavior was done across a 30 min period in which each rat was observed for 10 sec at 1 min intervals. Rats were observed and scored according to a scale as previously described [13,38], in which 0 was scored when the CFA treated hindpaw area was touching the mesh, and the area was blanched or distorted by the mesh; 1 was scored when the CFA treated hindpaw touched the mesh without blanching or distortion; 2 for the position when the CFA treated hindpaw was completely off of the mesh. For each hind paw, a cumulative score was obtained by adding the 30 scores during the 30 min testing period.

#### CPP Procedures

A single trial conditioning protocol was used for CPP as previously described [1]. All rats underwent a 3 day pre-conditioning habituation period with behavior recorded on day 3 to verify no pre-conditioning chamber preference. Analyses of the pre-conditioning (baseline) time spent in the conditioning chambers showed that rats spent equivalent time in the striped vs. the black walled chambers indicating no pre-existing chamber preference prior to counterbalancing further suggesting that any post-conditioning preferences observed reflect preference due to relief of ongoing pain, and not other potential factors such as anxiolytic effects of drug administration. On conditioning day, rats received the appropriate vehicle control paired with a chamber in the morning, and the appropriate drug treatment paired with the other chamber 4 hr later. Chamber pairings were counterbalanced. On test day, 20 hrs following the afternoon pairing, rats were placed in the CPP box with access to all chambers and behavior was recorded for 15 min for analysis for chamber preference.

#### Statistical Analysis

Effects of drug treatments on evoked pain were determined by 2-factor ANOVA for repeated measures with time serving as a within-subject factor. Differences from the post-treatment values were determined by Student-Neuman-Keuls *post-hoc *test. CPP data were analyzed before conditioning (baseline) and after conditioning using two-factor ANOVA (chambers vs. treatment) followed by Student's t-test with Bonferroni correction. No preconditioning differences in time spent in chambers between saline and CFA treated rats were observed, therefore baseline chamber data for each experiment was pooled across treatment. For all analyses, significance was set at p < 0.05.

## Competing interests

The authors declare that they have no competing interests.

## Authors' contributions

AO, MD, NE, JR, and RM: Performed the behavioral studies. MD and TK participated in the design of the study and performed the statistical analyses. TK and FP conceived of the study and participated in its design and coordination, and wrote and edited the manuscript. All authors read and approved the final manuscript.
